# Association Between *cnm*-Positive Streptococci and Cerebral Small Vessel Disease: Insights From Oral Health and Microbiome Status

**DOI:** 10.1016/j.identj.2026.109787

**Published:** 2026-08-01

**Authors:** Yujia Hao, Yuqiu Huang, Haoning Cai, Ruonan Chen, Xue Liang, Xiaojing Huang

**Affiliations:** a Clinical Research Center for Oral Tissue Deficiency Diseases of Fujian Province & Fujian Key Laboratory of Oral Diseases & Fujian Provincial Engineering Research Center of Oral Biomaterial, School and Hospital of Stomatology, Fujian Medical University, Fujian, China; b Stomatological Key laboratory of Fujian College and University & Institute of Stomatology & Research Center of Dental and Craniofacial Implants, School and Hospital of Stomatology, Fujian Medical University, Fujian, China

**Keywords:** *Streptococcus*, Cerebral small vessel disease, *cnm*, Oral health status, Oral microflora

## Abstract

**Introduction and aims:**

Cerebral small vessel disease (CSVD) is associated with various severe neurological outcomes; while oral *cnm*-positive streptococci are suggested to be involved in cerebrovascular lesions, the specific associative features between these bacteria and CSVD have not yet been systematically investigated. This study aims to investigate the prevalence of *cnm*-positive streptococci in patients with CSVD and explore the correlation between infection and CSVD severity. By integrating oral health indices and microbiome sequencing, we evaluate the oral hygiene status and microbial dysbiosis characteristics of *cnm*-positive streptococci carriers. Furthermore, *cnm*-positive streptococci derived from CSVD patients will be isolated, identified, and subjected to whole-genome sequencing to provide a foundation for future research.

**Methods:**

To explore *cnm*-positive streptococci prevalence and its association with CSVD, we conducted a case-control study comparing their oral detection rates between healthy controls and CSVD patients. We also performed 16S rRNA gene high-throughput sequencing of oral plaque microbiota and assessed oral health, including the simplified oral hygiene index (OHI-S), the decayed, missing, and filled teeth (DMFT) index, oral hygiene practices, gingival status, and saliva scores.

**Results:**

*cnm*-positive streptococci were more prevalent in CSVD patients, correlating with higher OHI-S and microbial dysbiosis. Multivariable regression models (adjusted for demographic/vascular risk factors) linked *cnm* positivity to periventricular hyperintensities (PVH), deep white matter hyperintensities (DWMH), Fazekas score, and total CSVD burden (not cerebral microbleeds (CMBs)/lacunes).

**Conclusion:**

Oral *cnm*-positive streptococci are independently associated with CSVD phenotypes, particularly those characterized by white matter injury. These findings presents a potential oral-cerebrovascular interaction and imply that managing specific virulent oral strains may be a noteworthy consideration in future clinical research.

## Introduction

Cerebral small vessel disease (CSVD) primarily affects small perforating arterioles and is characterized by lacunes, cerebral microbleeds (CMBs), cortical superficial siderosis, deep white matter hyperintensities (DWMH), enlarged perivascular spaces, and microinfarcts.^1^, ^2^, ^3^ CSVD contributes to roughly a quarter of ischemic strokes, serves as a major cause of intracerebral hemorrhage,^4^ and is linked to worse poststroke outcomes, cognitive impairment and dementia, depression, gait/mobility issues, and vascular dementia.^5^ Despite associations with traditional risk factors such as hypertension,^6^ arteriosclerosis,^7^ advanced age,^8^ lipohyalinosis,^9^ male sex^10^ and chronic kidney disease,^11^ CSVD in normotensive individuals highlights gaps in pathophysiology. Imaging studies have further indicated that the progression of CSVD can be nonlinear and may accelerate over time, suggesting a highly dynamic nature of its disease course.^12^^,^^13^ To manage CSVD, early risk factor intervention should be strengthened, centring on endothelial dysfunction and multidimensional phenotypic analysis, to provide a more scientific basis for prevention and treatment strategies.

In recent years, infectious and microbial factors have progressively entered the research landscape of cerebrovascular diseases. The oral cavity serves as a significant microbial reservoir in the human body, with nearly 700 bacterial species identified as capable of colonizing oral ecological niches,^14^ while also representing a common source of chronic low-grade inflammation.^15^^,^^16^ Its amenability to routine intervention has led to its consideration as a noteworthy, controllable upstream entry point for systemic vascular pathologies.

Invasive dental procedures and certain everyday oral activities can induce transient bacteraemia, thereby providing a potential hematogenous pathway for oral bacteria or their components to interact with the systemic vasculature.^17^^,^^18^ Among these, *Streptococcus* species represent notable members of the oral microbiota that exhibit a propensity for transient hematogenous translocation following mucosal barrier disruption.^19^, ^20^, ^21^, ^22^, ^23^ Accumulating evidence suggests that their potential systemic relevance may be dictated not by gross genus-level abundance, but by specific molecular virulence traits. Collagen-binding proteins represent pivotal virulence factors widely expressed across members of the genus *Streptococcus*, which fundamentally dictate their localized tissue adherence and pathogenic potential.^24^, ^25^, ^26^ For instance, the CpA protein in *Streptococcus pyogenes,*^27^ the CbpA protein in *Streptococcus gordonii,*^28^ and the Cbp40 protein in *Streptococcus suis*^29^ have all been characterized as critical determinants enabling bacteria to interact with the host extracellular matrix. Building upon this generic streptococcal virulence trait, this study specifically focused on the Cnm protein, a distinct collagen-binding protein originally identified in specific *Streptococcus mutans* (*S. mutans*) strains.^30^ The Cnm protein exhibits broad binding affinity to types I and IV collagen as well as laminin.^31^^,^^32^ In the context of cerebrovascular remodelling – where extracellular matrix components like collagen often become exposed or altered – circulating *cnm*-positive streptococci might theoretically exploit these compromised sites,^33^ potentially contributing to localized endothelial stress or blood-brain barrier impairment.^34^^,^^35^

Consequently, rather than focusing on gross microbial shifts at the entire genus level, evaluating specific functional virulence genes offers a more nuanced understanding of oral-cerebrovascular interactions.^36^^,^^37^ This case–control study assessed the prevalence of oral *cnm*-positive streptococci and evaluated their associations with CSVD neuroimaging severity.

## Materials and methods

### Participants

The G*Power software, version 3.1.9.2, was used to determine the sample size,^38^ with an anticipated *cnm*-positive streptococci prevalence of 20% in the experimental group and 7% in the control group. With a α-error of 5% and β-error of 20%, the final sample size was determined as 119 individuals. Between August 2024 and March 2025, we obtained consent for this study from 47 healthy subjects and 125 CSVD subjects who were admitted to Fujian Medical University’s first affiliated hospital. Participants with use of antibiotics, antimicrobial mouthwashes (eg, chlorhexidine), or probiotics within the past 3 months were excluded.^39^

All subjects underwent physical exams including brain magnetic resonance imaging (MRI) and cognitive function tests, and we also recorded traditional vascular risk factors such as hypertension, diabetes, dyslipidaemia (all medically diagnosed and/or on relevant medications), coagulation disorders, and a history of smoking or alcohol consumption (collected via interview on admission). Every procedure was carried out in compliance with the ethical principles outlined in the Declaration of Helsinki.

### Clinical check-up evaluations

The methodological assessment included standardized collection of clinical and lifestyle information: past medical history (stroke, hypertension, dyslipidaemia, diabetes and other cardiovascular diseases), receipt of relevant treatments and medication adherence, as well as alcohol intake and tobacco use. Comorbidities were classified using prespecified thresholds: diabetes was defined as haemoglobin A1c ≥ 6.5% and/or current use of antidiabetic medication^40^; dyslipidaemia as a previous diagnosis and/or triglycerides ≥ 150 mg/dL, high-density lipoprotein < 40 lt; 40 mg/dL, and/or low-density lipoprotein ≥ 140 mg/dL^41^; and hypertension as a previous clinical diagnosis and/or systolic blood pressure ≥ 140 mmHg and/or diastolic blood pressure ≥ 90 mmHg.^42^

### Dental evaluations

Dental evaluations and oral hygiene were assessed in a single visit by one calibrated dentist. Trained examiners performed oral assessments and were blinded to study-group assignments. We recorded the number of remaining teeth, caries experience, oral hygiene status, gingival index, saliva health index,^43^ and daily tooth-care habits by clinical examination. Caries experience was quantified using the decayed, missing, and filled teeth (DMFT) index in accordance with World Health Organization (WHO) criteria.^44^ Oral hygiene status was evaluated with the simplified oral hygiene index (OHI-S), which consists of the debris index-simplified (DI-S) and calculus index-simplified (CI-S) scored 0 to 3 on 6 index teeth for calculus coverage; the OHI-S total (0-6) is the sum of DI-S and CI-S and served as the individual oral hygiene metric.^45^

### Culture condition of streptococci

After being streaked onto selective Mitis-salivarius bacitracin agar that contained 15% sucrose and 100 U/mL of bacitracin (Sigma-Aldrich, St. Louis, MO, USA), oral specimens were incubated anaerobically at 37 °C for 48 h. Putative streptococci colonies were provisionally identified by colony morphology and subcultured in brain heart infusion broth (Difco/BD, Detroit, MI, USA), followed by anaerobic incubation at 37 °C for 24 h. For confirmation, Gram-stained smears were examined under a light microscope (40× objective; Olympus, Japan).

### *cnm* gene positivity assay

*cnm* primers reported by Nomura et al.^34^ were used (*cnm*-CF: CTG AGG TTA CTG TCG TTA AA and *cnm*-CR: CAC TGT CTA CAT AAG CAT TC; expected amplicon length 140 bp), and after retrieving and verifying the *cnm* reference sequence in NCBI, a recombinant plasmid containing the target fragment was synthesized. The plasmid was quantified using Qubit 3.0 (dsDNA HS Assay) and copy numbers were calculated according to the standard formula to prepare a 10-fold serial dilution ranging from 10^6^ to 10° copies per reaction, which was coamplified with samples in the same thermal cycler. Thirty cycles of 95 °C for 30 s, 60 °C for 30 s, and 72 °C for 1 min were used to thermocycle reactions using TaKaRa Ex Taq (Takara Bio), with a final extension at 72 °C for 7 min. Polymerase chain reaction (PCR) products were analysed by electrophoresis on 2% agarose gels; bands consistent with the insert size in the plasmid were deemed positive, purified using a gel extraction kit, and subjected to Sanger sequencing. The resulting sequences were queried by Basic Local Alignment Search Tool for nucleotides (BLASTn) against NCBI nt (core nucleotide) to verify whether the amplicons corresponded to the *cnm* gene. Participants were classified as *cnm*-positive if *cnm* gene was detected in any oral streptococcal isolate; otherwise, they were classified as *cnm*-negative.

### Microbiome 16S rRNA amplicon sequencing

Library construction and sequencing: The V3 to V4 hypervariable regions of the bacterial 16S rRNA gene were targeted for high-throughput sequencing. A 2-step PCR approach was employed for library construction. Briefly, genomic DNA was used as a template for the first round of PCR amplification using fusion primers 357F (5′-ACTCCTACGGRAGGCAGCAG-3′) and 806R (5′-GGACTACHVGGGTWTCTAAT-3′), which contained barcodes and partial sequencing adapters. PCR products were visualized via 1.2% agarose gel electrophoresis; samples exhibiting intact bands of the expected size were subjected to excision and purification from 2% agarose gels using the AxyPrep DNA Gel Extraction Kit (Axygen). The purified amplicons were quantified by fluorescence using the FTC-3000 Real-Time PCR system (Funglyn, Shanghai) and then pooled in equimolar ratios. A second round of PCR (8 cycles) was performed to attach Illumina-compatible adapters, sequencing primers, and dual-index tags to both ends of the target fragments. The final libraries were sequenced on the Illumina NovaSeq 6000 platform using the SP 500 Cycle Reagent Kit (Illumina) for PE250 paired-end sequencing at TinyGene Bio-Tech (Shanghai) Co., Ltd.

Bioinformatics and Statistical Analysis: Raw sequencing data were processed using Trimmomatic (v0.38) to filter low-quality reads, followed by adapter and primer removal using cutadapt (v2.6). Sequence processing, including denoising and chimera removal, was performed within the QIIME2 (v2020.8.0) environment using the q2-dada2 plugin to generate the Amplicon Sequence Variant (ASV) abundance table. Singleton ASVs were subsequently excluded. Taxonomic assignment was conducted against the SILVA 138 database.

To resolve the limitation of broad genus-level resolution and specifically handle compositional microbiome data issues, we implemented a more rigorous downstream analysis framework. First, relative abundance calculations were subjected to LEfSe (Linear Discriminant Analysis Effect Size) using Python (v2.7) to identify differentially abundant taxa, where nonparametric factorial Kruskal-Wallis tests were strictly coupled with a stringent False Discovery Rate (FDR) multiple-comparison correction (significance threshold: *P* < .05, LDA score ≥ 2.0). Second, to unmask features beyond the coarse genus level, the representative sequences of core differential streptococcal ASVs were extracted and subjected to high-precision local BLAST alignment against the NCBI RefSeq reference genome database to determine species-level pathogenic homology.

Alpha diversity indices (eg, Chao1, Shannon) were calculated using the vegan (v2.6-4) package in R (v3.6.3), and intergroup differences were evaluated using the compare_means function in ggpubr (v0.2.3). Regarding beta diversity, Bray–Curtis and Jaccard distance matrices were calculated based on ASV relative abundances using the vegdist function in vegan, while unweighted and weighted UniFrac distances were computed using phyloseq (v1.30.0). Principal Coordinate Analysis (PCoA) was performed using the ape (v5.7.1) package, and the statistical significance of community structural differences was assessed via ANOSIM in vegan. Visualizations were generated using ggplot2 (v3.4.2).

### Whole genome sequencing and de novo assembly

*Streptococcus vestibularis* (*S. vestibularis*) CSVD02 and *Streptococcus salivarius* (*S. salivarius*) CSVD05 were subcultured on brain heart infusion agar and incubated overnight. A sterile loop was used to select a single colony, which was then inoculated into 200 mL of brain heart infusion broth and incubated on a rotary shaker at 37 °C until reaching the logarithmic growth phase. The cultures were centrifuged at 12,000 rpm for 10 min at 4 °C, and the cell pellets were collected after discarding the supernatant. The pellets were washed with sterile water to remove residual medium components. Genomic DNA was extracted using the sodium dodecyl sulfate method. The extracted DNA was forwarded to Majorbio for whole genome sequencing and de novo assembly services. All analyses were performed via Majorbio Cloud (https://cloud.majorbio.com/).

For precise species classification, whole genome sequencing and de novo assembly data were analysed using FastANI to calculate pairwise average nucleotide identity (ANI) values between the 2 *cnm*-positive *streptococcal* isolates and 30 reference strains retrieved from the NCBI RefSeq database (including *S. vestibularis* and *S. salivarius*). An ANI threshold of ≥ 95% was applied for species delineation. To visualize the pairwise genomic similarity among all strains, an ANI heatmap was generated, in which red indicates high genomic similarity (>95%), whereas blue represents lower similarity (<95%).

### Imaging acquisition

Brain MRI was performed on 1.5 T/3.0 T Siemens scanners using a unified axial protocol (T1WI, T2WI, DWI/ADC, FLAIR, T2*-GRE; SWI additionally for microbleeds).^46^ Typical parameters: slice thickness 4 to 5 mm, interslice gap 0.5 to 1.0 mm, FOV 220 to 240 mm, matrix 256 × 256. Routine quality control was applied; scans with severe motion artifacts or missing key sequences were excluded.

### Imaging assessment

Lacunes: Subcortical 3 to 15 mm CSF-like cavities (hypointense on T1WI/FLAIR, hyperintense on T2WI) without mass effect; lesions with diffusion restriction or edema were not counted.^46^ DWMH: Deep white-matter hyperintensities on T2WI/FLAIR; extent judged by anatomical distribution and continuity, excluding periventricular caps/linings and U-fibres. PVH: Hyperintensities on T2WI/FLAIR directly abutting the lateral ventricular wall; evaluate periventricular distribution and extension into deep white matter, avoiding double-counting with DWMH.^47^ CMBs: Round/ovoid hypointense foci 2 to 5 mm (≤10 mm) with blooming on T2*-GRE/SWI; record location (lobar, deep, posterior fossa). Exclude vascular flow voids, calcification/mineralization, cavernous malformations, and artifacts.^48^ All neuroimaging markers were independently assessed by 2 trained raters blinded to clinical grouping and microbiological results. Inter-rater reliability was calculated based on the initial independent scores prior to consensus, with discrepancies resolved through joint review and consensus. Interrater correlation coefficients were 0.866 (95% CI: 0.823-0.899) for CMBs and 0.846 (95% CI: 0.797-0.884) for lacunae count. Kappa values were 0.853 (95% CI: 0.804-0.903) for DWMH and 0.861 (95% CI: 0.815-0.906) for PVH.

### Neuropsychological assessment

To comprehensively evaluate the participants’ neurological status, neuropsychological assessments were performed by an experienced neurologist blinded to the group assignments. The evaluation encompassed the following 3 dimensions:

Global cognitive function: The Mini-Mental State Examination (MMSE)^49^ and the Montreal Cognitive Assessment (MoCA)^50^ were employed to assess global cognitive proficiency.

Functional disability and activities of daily living: The modified Rankin Scale (mRS) was used to quantify the degree of neurological disability,^51^ while the Activities of Daily Living (ADL) Scale was utilized to evaluate physical independence and self-care capacity.^52^

Psychological status: The Hamilton Anxiety Scale (HAMA)^53^ and the Hamilton Depression Scale (HAMD)^54^ were administered to assess the participants’ psychological well-being and emotional distress.

### Statistical analyses

Data were processed using Excel 2019 and analysed with SPSS 26.0. The Kolmogorov–Smirnov test was used to determine whether continuous variables were normal. The *t*-test was used to compare normally distributed data, which were represented as mean ± SD (Welch’s *t*-test when variances were unequal); the Mann-Whitney U test was used to compare non-normally distributed data, which were displayed as median (P25, P75). Categorical variables were expressed as *n* (%) and compared by χ² or Fisher’s exact test, depending on expected frequencies. To evaluate the association between *cnm* carriage status and various CSVD imaging markers, multiple regression models were constructed. For the count data, including the number of CMBs and lacunes, Poisson regression models were employed to evaluate the associations. For ordinal data, such as PVH, DWMH, Fazekas scores, and total CSVD burden, ordinal logistic regression was performed. Multivariable models were constructed to adjust for potential confounders, including age, sex, body mass index (BMI), hypertension, dyslipidaemia, diabetes mellitus, smoking, alcohol consumption, history of cardiovascular disease, C-reactive protein (CRP) and glomerular filtration rate (GFR). Results are presented as relative risks (RRs) or odds ratios (ORs) with corresponding 95% confidence intervals (CIs). All statistical tests were 2-tailed, and a *P* < .05 was considered statistically significant.

## Results

### Subject’s characteristics

172 participants in all were enrolled and divided into 2 groups: the CSVD group (*n* = 125) and the health control (HC) group (*n* = 47). All individuals meeting the inclusion criteria provided consent and were included in the analysis set. Baseline characteristics for the overall cohort are summarized in Table 1, with group imbalances comprehensively evaluated using both *P*-values and standardized mean differences (SMDs). The CSVD group’s median age was 64 years old, accounting for 72.0% of males and 28.0% of females. The age median of HC group was 62 years, at a level of 57.45% of males and 42.55% of females. The 2 groups were tightly balanced in terms of BMI, hypertension, and diabetes (all |SMD| < 0.1 and *P* > .05). Mild-to-moderate imbalances were observed for sex (|SMD| = 0.31, *P* = .068), while substantial between-group differences (|SMD| ≥ 0.1 and *P* < .05) were noted for smoking status (|SMD| = 0.82 and *P* < .05), alcohol consumption (|SMD| = 0.55 and *P* < .05), dyslipidaemia (|SMD| = 0.25), history of ischemic stroke (|SMD| = 0.38), cerebrovascular disease attack frequency (|SMD| = 0.53), and related medication use. Notably, despite a nonsignificant *P*-value, age exhibited a profound baseline imbalance between the 2 groups (|SMD| = 2.21, *P* = .156).

**Table 1:** Subject’s characteristics.

Characteristic	CSVD (*n* = 125)	HC (*n* = 47)	*P* value	SMD
**Age, median [IQR]**	**64.0 (58.0, 72.0)**	**62.0 (54.0, 69.0)**	**.156**	**2.210**
**Female, *n* (%)**	**35.0 (28.0)**	**20.0 (42.6)**	**.068**	**0.310**
**BMI, median [IQR]**	**23.2 (22.4, 23.9)**	**23.2 (23.2, 23.2)**	**.950**	−**0.080**
**Risk factors, *n* (%)**				
Hypertension	75.0 (60.0)	28.0 (59.6)	.960	0.000
Dyslipidaemia	50.0 (40.0)	7.0 (14.9)	.002	0.250
Diabetes mellitus	18.0 (14.4)	6.0 (12.8)	.783	0.020
**Smoking status**			**<.001**	**0.820**
Never smoker	58.0 (46.40)	38.0 (80.85)		
Former smoker (quit ≥6 mo ago)	0.0 (0.00)	0.0 (0.00)		
Current smoker	32.0 (25.60)	2.0 (4.26)		
Recent quitter (quit <6 mo ago)	35.0 (28.00)	7.0 (14.89)		
**Alcohol consumption**			**.012**	**0.550**
Never drinker	68.0 (54.40)	36.0 (76.60)		
Light drinker	6.0 (4.80)	0.0 (0.00)		
Moderate drinker	6.0 (4.80)	2.0 (4.26)		
Heavy drinker	1.0 (0.80)	0.0 (0.00)		
Former drinker (currently abstinent)	44.0 (35.20)	9.0 (19.15)		
**History of cardiovascular disease**	**11.0 (8.8)**	**5.0 (10.6)**	**.940**	−**0.020**
**Medication history, *n* (%)**				
Antihypertensive medication (s)	72.0 (57.6)	4.0 (8.5)	<.001	0.490
Antiplatelet agent (s)	54.0 (43.2)	5.0 (10.6)	<.001	0.330
Anticoagulant (s)	2.0 (1.6)	3.0 (6.4)	.248	−0.050
Antidiabetic medication (s)	12.0 (9.6)	1.0 (2.1)	.184	0.070
**Laboratory examination, median [IQR]**				
Fibrinogen, g/L	2.9 (2.9, 2.9)	2.9 (2.9, 2.9)	.375	0.100
CRP, mg/L	1.2 (1.2, 1.7)	1.2 (1.2, 1.2)	.455	1.560
GFR, mL/min	88.4 (78.3, 95.9)	88.4 (88.4, 88.4)	.109	−4.160
**Stroke type**				
IS, *n* (%)	53.0 (42.4)	2.0 (4.3)	<.001	0.380
ICH, *n* (%)	10.0 (8.0)	0.0 (0.0)	.103	0.080
**Number of cerebrovascular events, median [IQR]**	**0.0 (0.0, 1.0)**	**0.0 (0.0, 0.0)**	**<.001**	**0.530**
**Neuroimaging features of CSVD, median [IQR]**				
Lacune count	0.0 (0.0, 2.0)	0.0 (0.0, 0.0)	<.001	1.990
PVH	2.0 (2.0, 3.0)	0.0 (0.0, 0.0)	<.001	2.150
CMBs	2.0 (0.0, 14.0)	0.0 (0.0, 0.0)	<.001	10.380
DWMH	2.0 (1.0, 3.0)	0.0 (0.0, 0.0)	<.001	1.850
Fazekas score	4.0 (3.0, 5.0)	0.0 (0.0, 0.0)	<.001	4.000
**Total CSVD burden, median [IQR]**	**2.0 (1.0, 4.0)**	**0.0 (0.0, 0.0)**	**<.001**	**2.160**
**Clinical scale scores, median [IQR]**				
MMSE score	23.0 (21.0, 25.0)	30.0 (30.0, 30.0)	<.001	−8.360
mRS score	1.0 (0.0, 1.0)	0.0 (0.0, 0.0)	<.001	0.950
MoCA score	14.0 (9.0, 20.0)	29.0 (29.0, 29.0)	<.001	−14.340
ADL score	100.0 (100.0, 100.0)	100.0 (100.0, 100.0)	.001	−1.800
HAMA score	4.0 (0.0, 11.0)	0.0 (0.0, 0.0)	<.001	6.400
HAMD score	6.0 (1.0, 11.0)	0.0 (0.0, 0.0)	<.001	7.090

ADL, activities of daily living; BMI, body mass index; CMBs, cerebral microbleed(s); CRP, C-reactive protein; CSVD, cerebral small vessel disease; DWMH, deep white matter hyperintensities; GFR, glomerular filtration rate; HAMA, Hamilton Anxiety Rating Scale; HAMD, Hamilton Depression Rating Scale; ICH, intracerebral haemorrhage; IQR, interquartile range; IS, ischemic stroke; MMSE, Mini-Mental State Examination; MoCA, Montreal Cognitive Assessment; mRS, modified rankin scale; PVH, periventricular hyperintensities.

Expectedly, profound variances (|SMD| > 1.0 and *P* < .001) were demonstrated across all neuroimaging markers, including the number of lacunes, CMBs, PVH, DWMH, Fazekas scores, and total CSVD burden. Within the CSVD group, the severity of white matter injury graded by the Fazekas score was distributed as follows: 31 patients scored 6 (24.8%), 28 scored 5 (22.4%), 31 scored 4 (24.8%), 16 scored 3 (12.8%), 1 scored 2 (0.8%), and 18 scored 0 (14.4%). Concurrently, highly significant differences were revealed across all clinical and functional scale scores (*P* ≤ .001). As illustrated in Table 1, the CSVD group exhibited significant cognitive decline compared with the HC group, characterized by noticeably lower scores on both the MMSE (*P* < .001) and MoCA (*P* < .001). Furthermore, patients with CSVD demonstrated mild neurological deficits, as evidenced by an elevated mRS score (*P* < .001). Regarding affective status, the CSVD group scored significantly higher on both the HAMA (*P* < .001) and HAMD (*P* < .001) relative to the healthy controls, indicating heightened levels of anxiety and depression. Although both groups maintained a median ADL score of 100.0, a statistically significant difference in daily functioning was still observed (*P* = .001).

### The detection of oral *cnm*-positive streptococci is associated with CSVD

The Cnm protein, a collagen-binding surface adhesin mainly expressed by streptococci,^55^ is associated with the occurrence and severity of CMBs.^56^^,^^57^ Given that oral streptococci are a primary reservoir for this protein, we sought to investigate its prevalence within our study cohort. Based on the baseline characteristics of subjects in the CSVD and HC groups summarized in the previous section, specific detection of *cnm*-positive streptococci was performed on the oral dental plaque of all subjects (Figure S1). To verify the reliability of these findings, the specific PCR products were confirmed by agarose gel electrophoresis and subjected to Sanger sequencing, with the resulting sequences deposited in the NCBI database. BLAST analysis demonstrated 100% coverage and 100% identity with the *cnm* reference sequence, thereby confirming the accuracy and specificity of the amplified fragments. Detection of *cnm*-positive streptococci in dental plaque samples revealed a higher prevalence in the CSVD group compared with the HC group [19.2% (24/125; 95% CI: 12.7%–27.2%) vs 2.1% (1/47; 95% CI: 0.0%–11.3%)]. Considering the asymmetrical sample distribution (HC: CSVD = 1: 2.6) and the low prevalence of *cnm*-positive streptococci in the HC group, we utilized Fisher’s exact test, which is specifically suited for small sample sizes or sparse data, and calculated 95% CIs to provide a more reliable estimate of effect size. Fisher’s exact test confirmed that the difference in detection rates between the 2 groups was statistically significant (*P* = .003). This distribution pattern suggests a preliminary association between *cnm*-positive streptococci and a higher prevalence in CSVD patients; however, these findings warrant further validation in more balanced cohorts.

To evaluate the clinical impact of oral *cnm*-positive streptococci carriage, we compared the severity of CSVD-related cerebral lesions and neuropsychological status between carriers and noncarriers (Table 2). Our findings indicated that *cnm* carriage was significantly associated with more severe neuroimaging manifestations, including exacerbated PVH, DWMH, Fazekas score, and total CSVD burden. Furthermore, these patients exhibited more profound impairments across multiple neuropsychological domains, characterized by declined cognitive function (MMSE/MoCA), compromised neurological status and activities of daily living (mRS/ADL), and aggravated affective states (HAMA/HAMD).

**Table 2:** Association between *cnm*-positive streptococci and the severity of CSVD.

Characteristic	CSVD-*cnm*-pos (*n* = 24)	CSVD-*cnm*-neg (*n* = 101)	*P* value
**Age, median [IQR]**	**67.0 (60.5, 75.0)**	**63.0 (57.0, 71.0)**	**.109**
**Female, *n* (%)**	**4.0 (16.7)**	**31.0 (30.7)**	**.169**
**BMI, median [IQR]**	**23.2 (23.2, 24.1)**	**23.2 (22.1, 23.7)**	**.256**
**Risk factors, *n* (%)**			
Hypertension	13.0 (54.2)	62.0 (61.4)	.516
Dyslipidaemia	10.0 (41.7)	40.0 (39.6)	.853
Diabetes mellitus	2.0 (8.3)	16.0 (15.8)	.536
**Smoking status**			**.244**
Never smoker	9.0 (37.5)	49.0 (48.5)	
Former smoker (quit≥6 mo ago)	0.0 (0.0)	0.0 (0.0)	
Current smoker	6.0 (25.0)	26.0 (25.7)	
Recent quitter (quit<6 mo ago)	9.0 (37.5)	26.0 (25.7)	
**Alcohol consumption**			**.393**
Never drinker	12.0 (50.0)	56.0 (55.5)	
Light drinker	0.0 (0.0)	6.0 (5.9)	
Moderate drinker	0.0 (0.0)	6.0 (5.9)	
Heavy drinker	1.0 (4.2)	0.0 (0.0)	
Former drinker (currently abstinent)	11.0 (45.8)	33.0 (32.7)	
**History of cardiovascular disease**	**2.0 (8.3)**	**9.0 (8.9)**	**1.000**
**Medication history, *n* (%)**			
Antihypertensive medication (s)	14.0 (58.3)	58.0 (57.4)	.936
Antiplatelet agent (s)	12.0 (50.0)	42.0 (41.6)	.454
Anticoagulant (s)	1.0 (4.2)	1.0 (1.0)	.348
Antidiabetic medication (s)	2.0 (8.3)	10.0 (1.0)	1.000
**Laboratory examination, median [IQR]**			
Fibrinogen, g/L	2.9 (2.8, 3.1)	2.9 (2.9, 2.9)	.714
CRP, mg/L	1.2 (1.0, 3.2)	1.2 (1.2, 1.5)	.979
GFR, mL/min	81.4 (73.8, 98.8)	88.4.0 (81.2, 95.4)	.212
**Stroke type**			
IS, *n* (%)	9.0 (37.5)	44.0 (43.7)	.589
ICH, *n* (%)	1.0 (4.2)	0.0 (8.9)	.725
**Number of cerebrovascular events, median[IQR]**	**0.0 (0.0, 1.0)**	**0.0 (0.0, 1.0)**	**.611**
**Neuroimaging features of CSVD, median[IQR]**			
Lacune count	0.0 (0.0, 4.5)	0.0 (0.0, 2.0)	.487
PVH	3.0 (2.0, 3.0)	2.0 (2.0, 3.0)	.022
CMBs	7.0 (3.5, 20.0)	0.0 (0.0, 11.0)	.001
DWMH	2.0 (2.0, 3.0)	2.0 (1.0, 2.0)	.002
Fazekas score	5.0 (4.0, 6.0)	4.0 (3.0, 5.0)	.004
**Total CSVD burden, median [IQR]**	**3.5 (2.0, 4.0)**	**2.0 (0.0, 4.0)**	**.001**
**Clinical scale scores, median [IQR]**			
MMSE score	21.0 (16.5.0, 21.0)	22.0 (21.0, 25.0)	.015
mRS score	1.0 (1.0, 2.0)	1.0 (0.0, 1.0)	<.001
MoCA score	11.0 (9.0, 13.5)	16.0 (9.0, 20.0)	<.010
ADL score	97.5.0 (85.0, 100.0)	100.00 (100.0, 100.0)	<.001
HAMA score	11.0 (4.0, 14.0)	4.0 (0.0, 9.0)	.003
HAMD score	10.0 (3.5, 19.0)	4.0 (0.0, 11.0)	.004

ADL, activities of daily living; BMI, body mass index; CMBs, cerebral microbleed(s); *cnm*-pos/neg, *cnm*-positive/negative; CRP, C-reactive protein; CSVD, cerebral small vessel disease; DWMH, deep white matter hyperintensities; GFR, glomerular filtration rate; HAMA, Hamilton Anxiety Rating Scale; HAMD, Hamilton Depression Rating Scale; ICH, intracerebral haemorrhage; IQR, interquartile range; IS, ischemic stroke; MMSE, Mini-Mental State Examination; MoCA, Montreal Cognitive Assessment; mRS, modified rankin scale; PVH, periventricular hyperintensities.

**Fig. 1:**
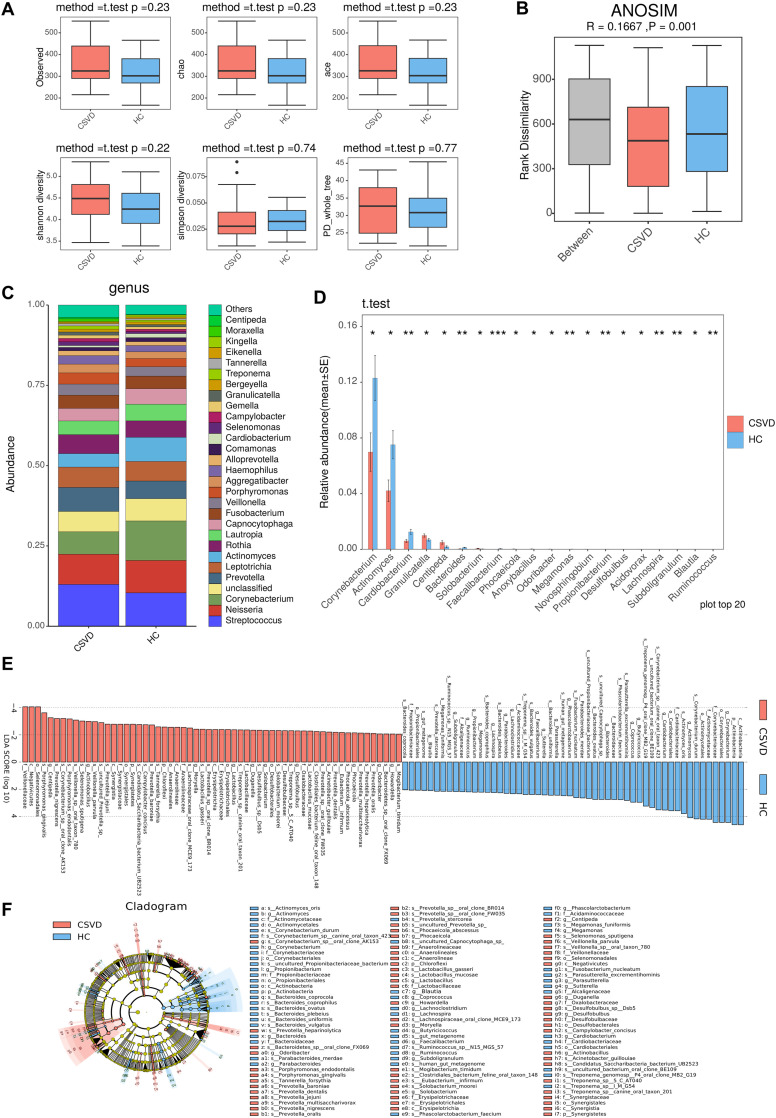
Oral microbiome differences in CSVD vs HC. (A) α-diversity: no group difference (*P* > .05). (B) β-diversity: clear separation (ANOSIM *R* = 0.2608, *P* = .001). (C) Genus-level profiles: Streptococcus more prominent in CSVD; Neisseria and Corynebacterium predominant in HC. (D) Streptococcus relative abundance: not statistically different. (E and F) LEfSe (LDA > 2.0) and cladogram: CSVD enriched for anaerobic proinflammatory and sulfate-reducing taxa; HC enriched for Actinobacteria, short-chain-fatty-acid producers, and diverse Bacteroides. CSVD, cerebral small vessel disease; HC, healthy controls; LEfSe, linear discriminant analysis effect size.

Furthermore, 16S rRNA gene high-throughput sequencing was performed to quantify the abundance of oral plaque microorganisms in all subjects, and the results showed that: The α-diversity of the CSVD and HC groups did not differ statistically significantly (*P* > .05) (Figure 1A). Conversely, β-diversity analysis using ANOSIM indicated a slight but highly significant separation in the microbial community structure between the CSVD and HC groups (*R* = 0.2608, *P* = .001), suggesting that the groups possess distinct microbial profiles despite some overlap (Figure 1B). At the genus level, *Streptococcus* and *Neisseria* constituted the predominant baseline taxa of the CSVD microbiota, while *Corynebacterium, Streptococcus*, and *Neisseria* were relatively prominent in the HC group (Figure 1C); however, according to the *t*-test analysis, there was no statistically significant difference in the relative abundance of *Streptococcus* between the 2 groups (Figure 1D). Furthermore, to identify differential species between the groups, linear discriminant analysis effect size (LEfSe) analysis was performed (LDA threshold > 2.0), and a Cladogram was used to visualize the phylogenetic relationships of significantly enriched species (Figure 1E and F). LEfSe analysis revealed significant distinct microbial signatures between the CSVD and HC groups. The HC group was predominantly enriched in *Actinobacteria*-related taxa, including *Corynebacterium, Corynebacteriaceae, Actinomycetaceae,* and *Actinomyces*, suggesting a relatively stable commensal microbiota profile in healthy controls. In contrast, the CSVD group exhibited an enrichment of various anaerobic and periodontal inflammation-associated taxa, such as *Prevotella, Porphyromonas gingivalis, Tannerella forsythia, Veillonellaceae, Selenomonadales, Treponema*, and *Solobacterium*. The cladogram further illustrated that the enriched taxa in the HC group were primarily clustered within the *Actinobacteria* lineage, whereas the CSVD group showed a broader distribution of enriched taxa across multiple anaerobic and inflammatory-related phylogenetic branches.

Although this study found no statistically significant difference in the genus-level abundance of *Streptococcus* between groups, this finding precisely constitutes the logical entry point of the present study. Genus-level taxonomic classification represents a relatively ‘coarse-grained’ level of resolution, encompassing multiple species and ASVs, and is therefore prone to an evident ‘aggregation effect’.^58^ Given the substantial functional heterogeneity among members of the genus *Streptococcus*, such aggregated analysis may dilute the pathogenic signal of specific highly virulent strains, such as *cnm*-positive strains, through the heterogeneity of other intrageneric members, thereby obscuring this signal in univariate testing.^59^^,^^60^ To address this and achieve a more granular perspective, we further stratified the CSVD patients into 2 subgroups based on the presence of the *cnm* virulence gene: the *cnm*-positive (CSVD-*cnm*-pos) and *cnm*-negative (CSVD-*cnm*-neg) groups. This approach aims to reveal, at the molecular level, the precise association between specific *Streptococcus* subgroups and the oral dysbiosis associated with CSVD.

**Fig. 2:**
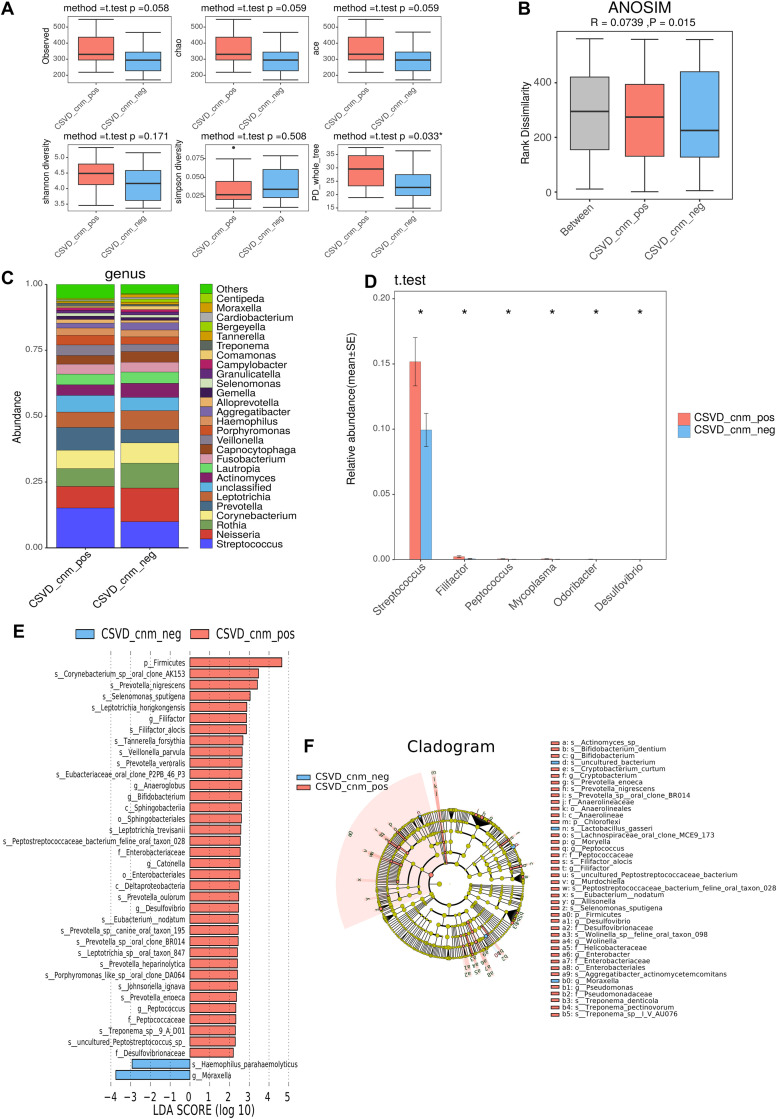
Oral microbiome shifts associated with *cnm*-positive carriage in CSVD. (A) Richness indices (Observed species, Chao1, ACE) were higher in CSVD-*cnm*-pos than CSVD-*cnm*-neg (α-diversity; *P* ≤ .005), while evenness (Shannon, Simpson) did not differ (*P* > .05). (B) β-diversity separated groups (ANOSIM *R* = 0.0722, *P* = .037). (C) Genus-level composition: CSVD-*cnm*-pos dominated by *Streptococcus*; CSVD-*cnm*-neg by *Neisseria*. (D) *Streptococcus* was significantly more abundant in CSVD-*cnm*-pos (*P* < .05). (E and F) LEfSe (LDA>2.0) and cladogram highlight enrichment of oral anaerobes/opportunistic pathogens in CSVD-*cnm*-pos versus common symbionts (eg, *Lactobacillus gasseri, Moraxella* spp.) in CSVD-*cnm*-neg. CSVD, cerebral small vessel disease; *cnm*-pos/neg, *cnm*-positive/negative.

Within the CSVD cohort, further stratification based on the carriage status of *cnm*-positive streptococci revealed that no significant differences were observed in α-diversity between the CSVD-*cnm*-pos and CSVD-*cnm*-neg groups (*P* > .05) (Figure 2A). β-diversity analysis further confirmed a statistically significant, albeit weak, separation in microbial community structure between the 2 groups (ANOSIM, *R* = 0.0739, *P* = .015) (Figure 2B). This indicates that while the overall community compositions of the CSVD-*cnm*-pos and CSVD-*cnm*-neg groups are highly similar, they remain distinct from one another. To explore the compositional differences at the genus level between CSVD-*cnm*-pos and CSVD-*cnm*-neg groups, a bar plot was used to compare the species composition at the genus level (Figure 2C). According to these findings, the CSVD-*cnm*-pos group was dominated by *Streptococcus*, while the CSVD-*cnm*-neg group was primarily composed of *Neisseria*. The bar plot *t*-test for the significantly different genera revealed that *Streptococcus* was significantly more abundant in the CSVD-*cnm*-pos group (*P* < .05) (Figure 2D). To further refine the identification of specific sequence variants driving the expansion of the genus *Streptococcus*, we tracked the candidate ASVs that displayed significant differential abundance between the *cnm*-positive and *cnm*-negative subcohorts within the CSVD population. Focusing on the abundant *Streptococcus* ASVs, sequence alignment verified that these enriched variants exhibited definitive genetic homology with reference *cnm*-positive *S. mutans* strains, specifically *S. mutans* LJ23 (GCF_000284575.1) and *S. mutans* OMZ175 (GCF_000339635.1), demonstrating sequence similarities of 84% to 100% and coverages of 65% to 100%. Collectively, these findings suggest that the aberrant elevation of *Streptococcus* abundance in the *cnm*-positive subcohort may be closely linked to the enrichment of these specific sequence variants sharing potential pathogenic phenotypic homology (Table S1). The CSVD-*cnm*-pos group was enriched with a greater number of discriminative taxonomic units. These included *Prevotella nigrescens, Prevotella veroralis, Selenomonas sputigena, Veillonella parvula, Leptotrichia hongkongensis, Filifactor alocis, Tannerella forsythia, Treponema, Peptococcus*, and *Desulfovibrio*. In contrast, the CSVD-*cnm*-neg group exhibited enrichment in only a limited number of taxa, primarily *Moraxella* and *Haemophilus parahaemolyticus* (Figure 2E and F). These findings suggest that the carriage of *cnm*-positive streptococci does not occur in isolation but is associated with a specific oral dysbiosis profile characterized by the enrichment of anaerobic, periodontitis-associated, and mature biofilm-related microbiota. This microbial co-occurrence status reflects a more inflammatory oral ecosystem in *cnm*-positive streptococci carriers.

### The potential role of oral hygiene status and *cnm*-positive streptococci colonization in CSVD pathogenesis

The oral hygiene status of patients with CSVD and HC group was evaluated using OHI-S. Based on OHI-S categories, the distribution differed markedly between groups: in the HC group, 70.21% had good hygiene (33/47), 29.79% fair (14/47), and 0.00% poor (0/47); in the CSVD group, 36.80% had good hygiene (46/125), 48.00% fair (60/125), and 15.20% poor (19/125). The between-group difference in OHI-S category distribution was significant (*z* = −4.223, *P* < .001). This finding indicates that oral hygiene problems in CSVD patients are characterized by a shift from ‘good’ to ‘fair/poor’ OHI-S categories, consistent with greater plaque/calculus accumulation and a significantly poorer overall oral hygiene status compared with healthy individuals. Even though there were no discernible differences between the 2 groups in self-reported oral hygiene behaviours such as the frequency of tooth brushing, the use of dental floss, mouthwashing habits, the OHI-S more accurately reflects the extent of dental plaque and calculus accumulation as an objective metric.

Additionally, the filled teeth index (FT) of the CSVD group was significantly higher than that of the HC group [0.00 (0.00, 1.00) vs 0.00 (0.00, 0.00), *z* = 2.479, *P* < .05]. The decayed, missing, and filled teeth index (DMFT), decayed teeth index (DT), and missing teeth index (MT) did not significantly differ between the CSVD and HC groups (Table 3). Rather than indicating active disease progression, the higher FT index in the CSVD group reflects a greater history of prior dental treatments for dental caries within this population. To clarify the impact of *cnm*-positive streptococci colonization on the oral health of patients with CSVD, this study further conducted a subgroup analysis of CSVD patients: compared with the CSVD-*cnm*-neg group, the DMFT index was significantly higher in the CSVD-*cnm*-pos group [7.50 (4.50, 13.00) vs 5.00 (3.00, 9.00), *z* = 2.240, *P* < .05], and this difference was mainly driven by the increase in the DT index – the DT index in the CSVD-*cnm*-pos group was significantly higher than that in the CSVD-*cnm*-neg group [4.00 (3.00, 5.00) vs 2.00 (0.00, 4.00), *z* = 2.110, *P* < .05] (Table 4).

**Table 3:** Oral conditions between HC and CSVD groups.

Characteristic	CSVD (*n* = 125)	HC (*n* = 47)	*P* value
**DT, median [IQR]**	**3.0 (1.0, 4.0)**	**2.0 (1.0, 4.0)**	**.766**
**MT, median [IQR]**	**1.0 (0.0, 4.0)**	**1.0 (0.0, 4.0)**	**.690**
**FT, median [IQR]**	**0.0 (0.0, 1.0)**	**0.0 (0.0, 0.0)**	**.013**
**DMFT, median [IQR]**	**6.0 (3.0, 9.0)**	**6.0 (3.0, 11.0)**	**.934**
**MFT, median [IQR]**	**2.0 (1.0, 5.0)**	**2.0 (0.0, 4.0)**	**.867**
**FDP, median [IQR]**	**2.0 (0.0, 8.0)**	**0.0 (0.0, 3.0)**	**.016**
**DI-S, median [IQR]**			**.006**
No debris	5.0 (4.0)	0.0 (0.0)	
Debris covering <1/3 of tooth surface	65.0 (52.0)	39.0 (83.0)	
Debris covering 1/3 to 2/3 of tooth surface	41.0 (32.8)	6.0 (12.8)	
Debris covering >2/3 of tooth surface	14.0 (11.2)	2.0 (4.3)	
**CI-S, median [IQR]**			**<.001**
No calculus	56.0 (44.8)	41.0 (87.2)	
Calculus covering <1/3 of tooth surface	52.0 (41.6)	6.0 (12.8)	
Calculus covering 1/3 to 2/3 of tooth surface	15.0 (12.0)	0.0 (0.0)	
Calculus covering >2/3 of tooth surface	2.0 (1.6)	0.0 (0.0)	
**OHI-S, median [IQR]**			**<.001**
Good oral hygiene	46.0 (36.8)	33.0 (70.2)	
Fair oral hygiene	60.0 (48.0)	14.0 (29.8)	
Poor oral hygiene	19.0 (15.2)	0.0 (0.0)	
**Toothbrushing frequency, times/day, median [IQR]**	**2.0 (1.0, 2.0)**	**2.0 (2.0, 2.0)**	**.029**
**Dental floss use, *n* (%)**			**.024**
No	87.0 (69.6)	24.0 (51.1)	
Yes	38.0 (30.4)	23.0 (48.9)	
**Mouthwash use, *n* (%)**			**.009**
No	65.0 (52.0)	14.0 (29.8)	
Yes	60.0 (48.0)	33.0 (70.2)	
**Saliva score, *n* (%)**			**.008**
Changed	40.0 (32.0)	27.0 (57.5)	
Unhealthy	62.0 (49.6)	13.0 (27.7)	
Healthy	23.0 (18.4)	7.0 (14.9)	
**Gingival index, *n* (%)**			**.034**
Normal	81.0 (64.8)	40.0 (85.1)	
Redness–swelling	37.0 (29.6)	6.0 (12.8)	
Bleeding	7.0 (5.6)	1.0 (2.2)	

CI-S, calculus index–simplified; DI-S, debris index–simplified; DMFT, decayed-missing-filled teeth index; DT, decayed teeth; FDP, fixed dental prosthesis; FT, filled teeth; IQR, interquartile range; MFT, missing and filled teeth; MT, missing teeth; OHI-S, simplified oral hygiene index.

**Table 4:** Oral health status between CSVD *cnm*-negative and CSVD *cnm*-positive groups

Characteristic	CSVD *cnm*-pos (*n* = 24)	CSVD *cnm*-neg (*n* = 101)	*P* value
**DT, median [IQR]**	**4.0 (3.0, 5.0)**	**2.0 (0.0, 4.0)**	**.035**
**MT, median [IQR]**	**2.5 (1.0, 7.5)**	**1.0 (0.0, 3.0)**	**.251**
**FT, median [IQR]**	**1.0 (0.0, 1.0)**	**0.0 (0.0, 1.0)**	**.432**
**DMFT, median [IQR]**	**7.5 (4.5, 13.0)**	**5.0 (3.0, 9.0)**	**.025**
**MFT, median [IQR]**	**3.0 (1.0, 8.0)**	**2.0 (0.0, 4.0)**	**.233**
**FDP, median [IQR]**	**2.0 (2.0, 10.5)**	**1.0 (0.0, 8.0)**	**.023**
**DI-S, median [IQR]**			**<.001**
No debris	0.0 (0.00)	5.0 (4.95)	
Debris covering <1/3 of tooth surface	0.0 (0.00)	65.0 (64.36)	
Debris covering 1/3 to 2/3 of tooth surface	18.0 (75.00)	23.0 (22.77)	
Debris covering >2/3 of tooth surface	6.0 (25.00)	8.0 (7.92)	
**CI-S, median [IQR]**			**<.001**
No calculus	0.0 (0.00)	56.0 (55.45)	
Calculus covering <1/3 of tooth surface	17.0 (70.83)	35.0 (34.65)	
Calculus covering 1/3 to 2/3 of tooth surface	5.0 (20.83)	10.0 (9.90)	
Calculus covering >2/3 of tooth surface	2.0 (8.33)	0.0 (0.00)	
**OHI-S, median [IQR]**			**<.001**
Good oral hygiene	0.0 (0.00)	46.0 (45.54)	
Fair oral hygiene	14.0 (58.33)	46.0 (45.54)	
Poor oral hygiene	10.0 (41.67)	9.0 (8.91)	
**Toothbrushing frequency, times/day, median [IQR]**	**1.0 (1.0, 1.0)**	**2.0 (1.0, 2.0)**	**<.001**
**Dental floss use, *n* (%)**			**.034**
No	21.0 (87.5)	66.0 (65.4)	
Yes	3.0 (12.5)	35.0 (34.7)	
**Mouthwash use, *n* (%)**			**.012**
No	18.0 (75.0)	47.0 (46.5)	
Yes	6.0 (25.0)	54.0 (53.5)	
**Saliva score, *n* (%)**			**.015**
Changed	8.0 (33.3)	32.0 (31.7)	
Unhealthy	7.0 (29.2)	55.0 (54.5)	
Healthy	9.0 (37.5)	14.0 (13.9)	
**Gingival index, *n* (%)**			**<.001**
Normal	5.0 (20.8)	76.0 (75.3)	
Redness–swelling	14.0 (58.3)	23.0 (22.8)	
Bleeding	5.0 (20.8)	2.0 (2.0)	

CI-S, calculus index–simplified; *cnm*-pos/neg, *cnm*-positive/negative; DI-S, debris index–simplified; DMFT, decayed–missing–filled teeth index; DT, decayed teeth; FDP, fixed dental prosthesis; FT, filled teeth; IQR, interquartile range; MFT, missing and filled teeth; MT, missing teeth; OHI-S, simplified oral hygiene index.

Comprehensive analysis of the above results shows that the oral health abnormalities in CSVD patients present a multifaceted profile. The general CSVD population exhibits poorer current oral hygiene (manifested by the shift in OHI-S classification) and a more extensive history of dental intervention (manifested by the increase in the FT index). Notably, active and ongoing dental caries burdens (manifested by the increases in DMFT and DT indices) are intimately linked to the specific colonization status of *cnm*-positive streptococci rather than CSVD status alone. These differences in oral health indicators suggest that both the colonization of *cnm*-positive streptococci and the pathological state of CSVD are closely intertwined with oral hygiene maintenance, which may subsequently disrupt the oral microflora balance and potentially elevate the risk of systemic vascular diseases.

### Regression analyses with *cnm*-positive streptococci detection as the independent variable

Within the CSVD group, Poisson regression models were employed to evaluate the associations between oral *cnm*-positive streptococci carriage status and CMBs as well as lacunes (Table 5). In the unadjusted model, no statistically significant association was observed between oral *cnm*-positive streptococci carriage status and either CMBs (RR = 1.529, 95% CI: 0.884-2.647, *P* > .05) or lacunes (RR = 1.031, 95% CI: 0.556-1.922, *P* > .05). In Model 1, which adjusted for age and sex, the associations remained statistically nonsignificant for both CMBs (RR = 1.560, 95% CI: 0.932-2.612, *P* > .05) and lacunes (RR = 1.098, 95% CI: 0.583-2.067, *P* > .05). Furthermore, after fully adjusting for age, sex, BMI, hypertension, dyslipidaemia, diabetes mellitus, smoking, alcohol consumption, history of cardiovascular disease, CRP and GFR in Model 2, the independent association of oral *cnm*-positive streptococci carriage status with CMBs (RR = 1.505, 95% CI: 0.827-2.598, *P* > .05) or lacunes (RR = 1.117, 95% CI: 0.574-2.173, *P* > .05) remained nonsignificant.

**Table 5:** Association of *cnm*-positive streptococci with CMBs and lacunes in a Poisson regression analysis.

Variable	Unadjusted		Model 1		Model 2	
	RR(95% CI)	*P*	RR(95% CI)	*P*	RR(95% CI)	*P*
**CMBs**	**1.529 (0.884, 2.647)**	**.129**	**1.560 (0.932, 2.612)**	**0.091**	**1.505 (0.872, 2.598)**	**.142**
**Lacune count**	**1.031 (0.556, 1.911)**	**.923**	**1.098 (0.583, 2.067)**	**0.772**	**1.117 (0.574, 2.173)**	**.745**

CMBs, cerebral microbleed(s).

Unadjusted: No confounding factors were adjusted.

Model 1: Adjusted for age and sex.

Model 2: Adjusted for age, sex, body mass index (BMI), hypertension, dyslipidaemia, diabetes mellitus, smoking, alcohol consumption, history of cardiovascular disease, C-reactive protein (CRP) and glomerular filtration rate (GFR).

Within the CSVD group, ordinal logistic regression analysis was performed with oral *cnm*-positive streptococci carriage status as the independent variable and PVH, DWMH, Fazekas score, and total CSVD burden as the dependent variables (Table 6). In the unadjusted model, oral *cnm*-positive streptococci carriage status exhibited significant positive associations with multiple CSVD neuroimaging phenotypes and overall disease severity (all *P* < .05). The specific statistical parameters were as follows: PVH (OR = 2.694, 95% CI: 1.085-6.693, *P* < .05), DWMH (OR = 3.522, 95% CI: 1.480-8.381, *P* < .05), Fazekas score (OR = 3.037, 95% CI: 1.332-6.931, *P* < .05), and total CSVD burden (OR = 3.677, 95% CI: 1.582-8.542, *P* < .05). In Model 1, which adjusted for age and sex, oral *cnm*-positive streptococci carriage status remained significantly associated with DWMH, Fazekas score, and total CSVD burden; although a positive trend was maintained for PVH, its statistical significance was lost in this model [PVH: OR = 2.474, 95% CI: 0.976-6.271, *P* > .05; DWMH: OR = 3.317, 95% CI: 1.379-7.988, *P* < .05; Fazekas score: OR = 2.924, 95% CI: 1.266-6.753, *P* < .05; total CSVD burden: OR = 3.380, 95% CI: 1.445-7.917, *P* < .05]. Furthermore, after fully adjusting for age, sex, BMI, hypertension, dyslipidaemia, diabetes mellitus, smoking, alcohol consumption, history of cardiovascular disease, CRP and GFR in Model 2, oral *cnm*-positive streptococci carriage status emerged as an independent risk factor for all imaging severity indicators, with its statistical significance fully re-emerging and becoming further pronounced (all *P* < .05) [PVH: OR = 2.782, 95% CI: 1.065-7.265, *P* < .05; DWMH: OR = 3.515, 95% CI: 1.418-8.715, *P* < .05; Fazekas score: OR = 3.203, 95% CI: 1.357-7.561, *P* < .05; total CSVD burden: OR = 4.133, 95% CI: 1.692-10.105, *P* < .05].

**Table 6:** Association of *cnm*-positive streptococci with PVH, DWMH, Fazekas score and total CSVD burden in an ordinal logistic regression analysis.

Variable	Unadjusted		Model 1		Model 2	
	OR(95% CI)	*P*	OR(95% CI)	*P*	OR(95% CI)	*P*
**PVH**	**2.694 (1.085, 6.693)**	**.033**	**2.474 (0.976, 6.271)**	**.056**	**2.782 (1.065, 7.265)**	**.037**
**DWMH**	**3.522 (1.480, 8.381)**	**.004**	**3.317 (1.379, 7.988)**	**.007**	**3.515 (1.418, 8.715)**	**.007**
**Fazekas score**	**3.037 (1.332, 6.931)**	**.008**	**2.924 (1.266, 6.753)**	**.012**	**3.203 (1.357, 7.561)**	**.008**
**Total CSVD burden**	**3.677 (1.582, 8.542)**	**.002**	**3.380 (1.445, 7.917)**	**.005**	**4.133 (1.692, 10.105)**	**.002**

CSVD, cerebral small vessel disease; DWMH, deep white matter hyperintensities; PVH, periventricular hyperintensities.

Unadjusted: No confounding factors were adjusted.

Model 1: Adjusted for age and sex.

Model 2: Adjusted for age, sex, body mass index (BMI), hypertension, dyslipidaemia, diabetes mellitus, smoking, alcohol consumption, history of cardiovascular disease, C-reactive protein (CRP) and glomerular filtration rate (GFR).

### Taxonomic identification and whole genome sequencing characterization of representative *cnm*-positive streptococcal isolates

**Fig. 3:**
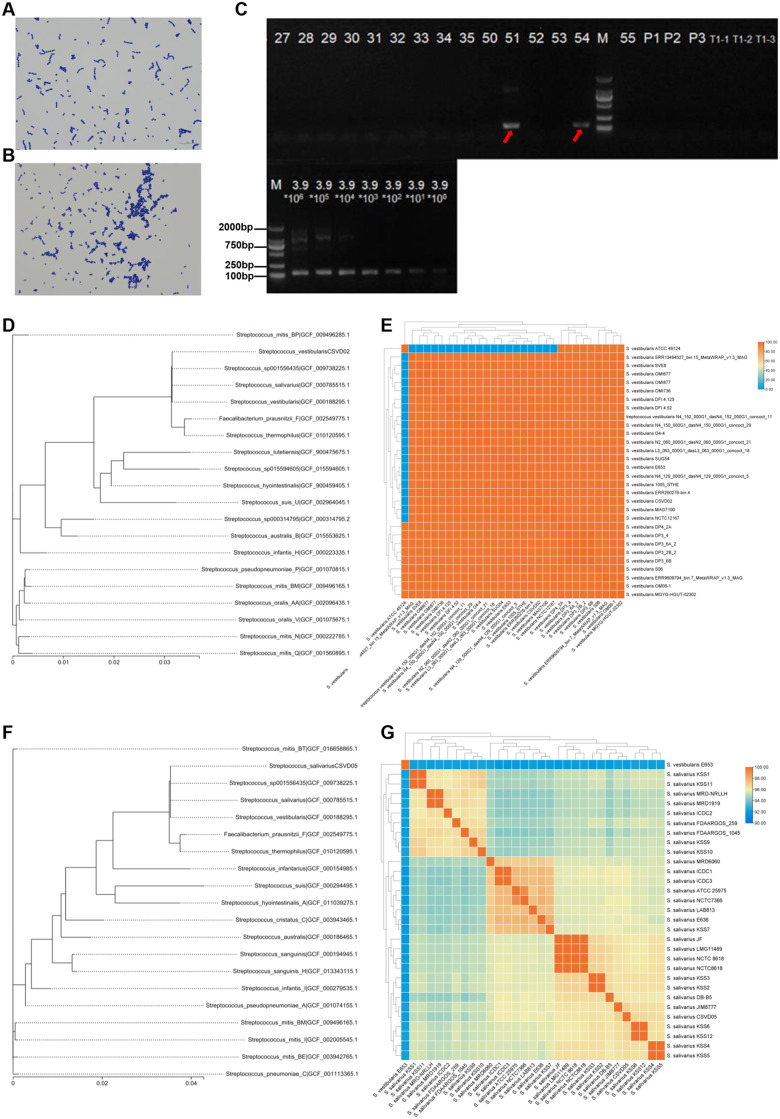
Phenotypic and genomic confirmation of *cnm*-positive isolates. (A) Gram-stained image of *S. vestibularis* CSVD02. (B) Gram-stained image of *S. salivarius* CSVD05. (C) *cnm*-PCR shows expected bands in both isolates (Lane 51: *S. vestibularis* CSVD02; lane 54: *S. salivarius* CSVD05); recombinant *cnm* plasmid (+) and NTC (−) validate the assay. Amplicons Sanger-sequenced and deposited in NCBI; BLAST 100% coverage/identity. (D) 16S phylogeny places CSVD02 proximate to *S. vestibularis* but not strictly monophyletic; branch distances favor *S. vestibularis* over *S. salivarius*. (E) FASTANI vs 30 *S. vestibularis* RefSeq genomes: all ANI > 95% → CSVD02 confirmed as *S. vestibularis*. (F) Within the *Salivarius* group, 16S topology is unstable (≥ 99% identity), limiting resolution for CSVD05. (G) FASTANI vs 30 *S. salivarius* RefSeq genomes: all ANI > 95% → CSVD05 confirmed as *S. salivarius*. ANI, average nucleotide identity; BLAST, Basic Local Alignment Search Tool; NTC, no-template control; RefSeq, NCBI Reference Sequence.

To clarify the genetic background of *cnm*-positive streptococci and contribute to subsequent mechanistic research, this study screened 2 representative *cnm*-positive isolates from dental plaque samples of CSVD patients, which were designated as *S. vestibularis* CSVD02 and *S. salivarius* CSVD05. Phenotypic identification and molecular identification were performed for these 2 representative isolates, followed by whole-genome sequencing. Gram staining revealed that *S. vestibularis* CSVD02 and *S. salivarius* CSVD05 both appeared as Gram-positive streptococci arranged in pairs or short chains (Figure 3A and B). In addition, specific PCR targeting the *cnm* gene produced amplicons of the expected size in both isolates, with a recombinant *cnm* plasmid serving as the positive control and the no-template control showing no amplification (Figure 3C). The amplicons were subsequently subjected to Sanger sequencing and deposited in the NCBI database; BLAST analysis demonstrated 100% coverage and 100% identity with the reference sequence, confirming the specificity and accuracy of the assay. Collectively, these results supported both the phenotypic features of the isolates and the presence of the *cnm* gene, thereby establishing a preliminary framework for subsequent 16S rRNA phylogenetic analysis and ANI-based species delineation.

The 16S rRNA–based phylogenetic tree, which included other *Streptococcus* species to confirm genus-level classification, showed that the clinical isolate *S. vestibularis* CSVD02 did not form a monophyletic clade with the reference strain *S. vestibularis* (GCF_000188295.1) (Figure 3D). However, branch distance analysis indicated that *S. vestibularis* CSVD02 was closer to *S. vestibularis* than to *S. salivarius*. Previous studies have demonstrated that, based on 16S rRNA gene sequences, streptococci can be divided at the species level into eight major groups, including the *Salivarius* group.^61^ Nevertheless, the 16S rRNA sequences of *S. vestibularis* and *S. salivarius* generally share more than 99% similarity, and phylogenetic trees often fail to reliably distinguish between the 2 species, with frequent topological intermingling.^62^^,^^63^ To overcome these limitations, we further employed ANI analysis, which has become the gold standard for microbial species delineation, with a threshold of ≥ 95% indicating the same species. We calculated the ANI between *S. vestibularis* CSVD02 and 30 reference strains of *S. vestibularis* available in the NCBI RefSeq database to quantify their genomic similarity. Using FASTANI, we also generated a comparative ANI heatmap, where red cells denote ANI values > 95% and blue cells denote values < 95% (Figure 3E). The results showed that all ANI values between *S. vestibularis* CSVD02 and the reference strains exceeded 95%, thereby providing support for its classification as *S. vestibularis*.

In the 16S rRNA-based phylogenetic tree (Figure 3F), although the isolate *S. salivarius* CSVD05 appeared closer in sequence distance to *S. vestibularis*, the high conservation of 16S rRNA among *Salivarius* group members and the unstable topology of 16S-based phylogenies necessitated further validation. ANI analysis demonstrated that *S. salivarius* CSVD05 shared ANI values exceeding 95% with 30 *S. salivarius* reference strains in the NCBI RefSeq database, thus supporting its identification as *S. salivarius* (Figure 3G).

**Fig. 4:**
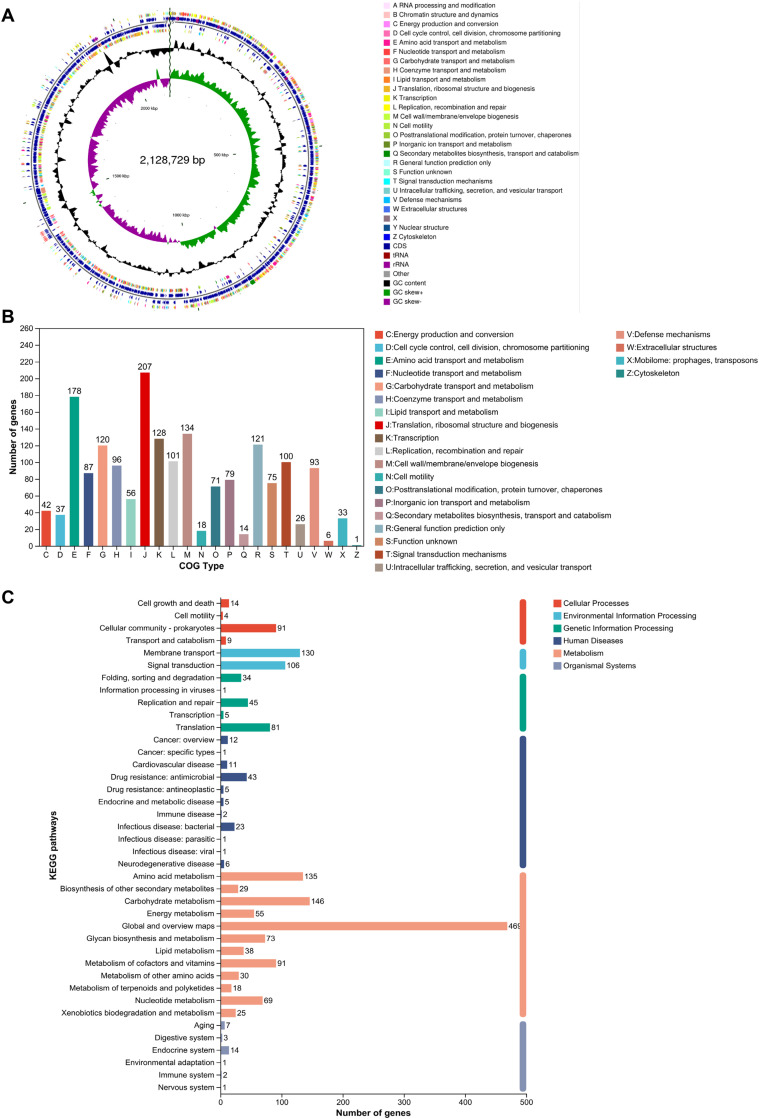
Genome architecture and functional landscape of *S. vestibularis* CSVD02. (A) Single circular chromosome (1,906,948 bp; GC 67.65%). (B) COG annotation enriched in translation/ribosomal structure/biogenesis (203 genes), amino acid transport/metabolism (173), and cell wall/membrane/envelope biogenesis (113). (C) KEGG pathways broadly cover cellular processes, environmental information processing, human diseases, metabolism, and organismal systems. COG, Clusters of Orthologous Groups; GC, guanine–cytosine content; KEGG, Kyoto Encyclopedia of genes and genomes.

**Fig. 5:**
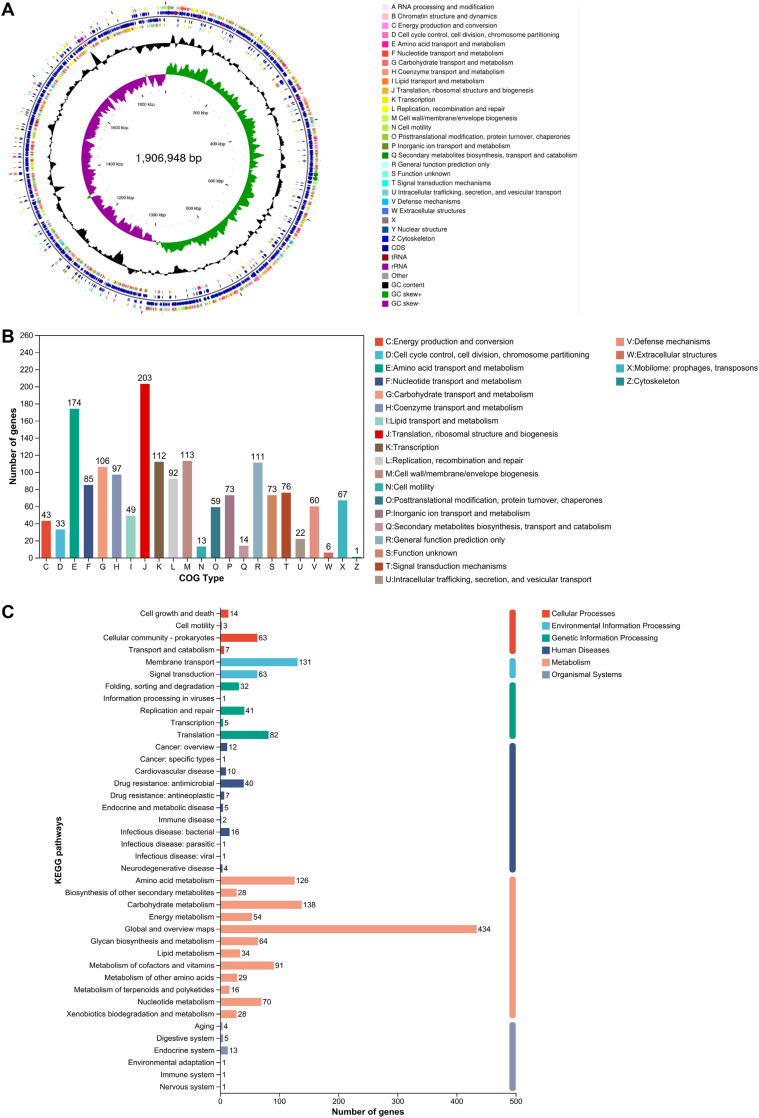
Genome architecture and functional landscape of *S. salivarius* CSVD05. (A) Single circular chromosome (2,128,729 bp; GC 67.67%). (B) COG annotation enriched in translation/ribosomal structure/biogenesis (207 genes), amino acid transport/metabolism (178), and cell wall/membrane/envelope biogenesis (134). (C) KEGG pathways span cellular processes, environmental information processing, human diseases, metabolism, and organismal systems. COG, Clusters of Orthologous Groups; GC, guanine–cytosine content; KEGG, Kyoto Encyclopedia of Genes and Genomes.

Next, we performed whole-genome sequencing on *S. vestibularis* CSVD02 and *S. salivarius* CSVD05 isolates carrying the *cnm* gene. The results revealed that both isolates consist of a single circular chromosome. The genome of *S. vestibularis* CSVD02 has a total length of 1,906,948 bp with an average GC content of 67.65%, while that of *S. salivarius* CSVD05 comprises 2,128,729 bp with an average GC content of 67.67% (Figures 4A and 5A). Functional annotation based on the Clusters of Orthologous Groups of Proteins (COG) database showed that the genes in both isolates were primarily associated with the following categories: Translation, ribosomal structure and biogenesis (203 genes in *S. vestibularis* CSVD02 and 207 genes in *S. salivarius* CSVD05), amino acid transport and metabolism (173 genes in *S. vestibularis* CSVD02 and 178 genes in *S. salivarius* CSVD05), and cell wall/membrane/envelope biogenesis (113 genes in *S. vestibularis* CSVD02 and 134 genes in *S. salivarius* CSVD05) (Figures 4B and 5B). According to Kyoto Encyclopedia of Genes and Genomes (KEGG) pathway analysis, both genomes are engaged in a variety of pathways, such as metabolism, organismal systems, human diseases, environmental information processing, and cellular processes. Overall, although the 2 isolates differ in genome size, their functional annotation profiles are highly consistent, suggesting significant commonality in their genus-level conserved functional framework and metabolic network (Figures 4C and 5C).

## Discussion

We acknowledge the imbalance in sample sizes between the HC and CSVD groups (ratio of 1: 2.6) and the limited number of *cnm*-positive cases in the HC group, which could inherently introduce statistical challenges. To mitigate this potential bias and ensure statistical rigor, Fisher’s exact test was initially employed, and 95% confidence intervals were reported. Furthermore, recognizing that conventional *P*-values can be sensitive to sample size discrepancies, we comprehensively calculated SMDs to transparently evaluate baseline imbalances. Although several parameters exhibited statistical imbalances (|SMD| ≥ 0.1), such as sex (|SMD| = 0.31) and age (|SMD| = 2.21), we fully neutralized these confounding effects by constructing a comprehensive, tiered regression model. All potentially imbalanced and established risk factors – including age, sex, BMI, hypertension, dyslipidaemia, diabetes mellitus, smoking, alcohol consumption, history of cardiovascular disease, CRP and GFR – were strictly adjusted as covariates. Notably, the pathological association between oral *cnm*-positive streptococci carriage and the severity of CSVD white matter hyperintensities remained robust and stable across all levels of adjustment. Therefore, despite the unequal sample sizes, our stringent statistical control over these comprehensive clinical confounders substantially enhances the reliability of our findings and minimizes the likelihood of potential bias.

A previous retrospective study involving 111 stroke patients reported that the detection rate of *cnm*-positive streptococci among the 111 stroke patients was 19%.^64^ In this study, the detection rate of *cnm*-positive streptococci in a cohort of 125 patients with CSVD reached 19.2%, comparable to the data from the aforementioned study.

The regression analysis in this study demonstrated a significant statistical association between *cnm*-positive streptococci and several CSVD phenotypes, including PVH, DWMH, Fazekas score, and total CSVD burden. While our cross-sectional data cannot determine the temporal sequence or causal direction of these relationships, these findings may be discussed in light of existing biological hypotheses. For instance, previous literature has highlighted the reciprocal relationship between blood-brain barrier (BBB) injury and white matter hyperintensities.^65^^,^^66^ Given that *cnm*-positive streptococci have been reported to adhere to injured cerebral endothelial cells and affect BBB permeability in experimental models,^67^ it is theoretically plausible that *cnm*-related endothelial interactions might co-occur with or parallel the BBB dysfunction observed in CSVD. Additionally, from a systemic perspective, oral dysbiosis-associated chronic inflammation has been hypothesized to correlate with large-artery stiffness or atherosclerosis, which could potentially alter downstream cerebral perfusion.^68^, ^69^, ^70^ However, we must emphasize that because this study lacks longitudinal follow-up, we cannot rule out reverse causation; it is equally possible that CSVD-induced neurological disability or systemic inflammatory states led to poorer oral hygiene and a subsequent increase in *cnm*-positive strains. Therefore, these integrative pathways remain strictly hypothetical. Longitudinal studies and prospective cohorts are indispensable to elucidate whether oral *cnm*-positive streptococci actively contribute to CSVD progression or merely serve as a biomarker of advanced disease and neglected oral health. The significance of this study lies in: (1) Even after adjusting for age, sex, and cardiometabolic risk factors, *cnm*-positive streptococci carrier status remained independently associated with PVH, DWMH, Fazekas score, and total CSVD burden. This association suggests that *cnm*-positive streptococci carrier status is consistently linked to a CSVD phenotype predominantly characterized by white matter injury, which parallels the known biological processes of diffuse white matter changes characterized by ‘chronic endothelial dysfunction, mild blood-brain barrier leakage, and low-grade inflammation/perfusion impairment’. Concurrently, this study also has limitations: (1) This study currently only provides statistical cross-sectional associations linking CSVD and *cnm*-positive streptococci. The underlying pathways in CSVD await mechanistic validation, and related experiments are underway, with data not yet published. (2) The case-control design limits inferences about causality and temporality. Future validation in longitudinal cohorts and intervention trials is necessary to clarify whether *cnm*-positive streptococci act as an independent risk marker, a confounding factor, or a reverse-causational outcome in CSVD, and to evaluate the actual relationship between oral microecological interventions and CSVD clinical outcomes.

From a perspective of future research directions, these preliminary findings raise the open question of whether dental risk assessment might eventually benefit from the evaluation of specific virulence genes, rather than genus-level abundance profiling alone.^36^^,^^37^ For individuals colonized by strains carrying collagen-binding proteins, such as Cnm and Cbm,^71^ future prospective studies are needed to evaluate whether targeted oral biofilm management could carry implications for systemic health. For individuals colonized by high-risk strains carrying collagen-binding proteins, such as Cnm, the establishment of an ‘intensified prevention and intervention protocol’ is recommended. Prior to dental procedures with a high likelihood of inducing transient bacteremia,^72^ including tooth extraction and deep periodontal scaling/root planing,^73^ infection control should be rigorously implemented through preoperative antimicrobial mouth rinses and minimally invasive operative techniques.^73^ In parallel, periodontal maintenance intervals should be shortened, for example to every 3 months, and plaque control should be reinforced.^74^ These measures may be combined with antiadhesion strategies, such as competitive inhibitors, or targeted antimicrobial interventions against strains harbouring collagen-binding protein genes, with the aim of reducing their colonization burden and risk of hematogenous dissemination. Furthermore, although highly speculative at this stage, these data provide a conceptual rationale for further discussing the long-term feasibility of interdisciplinary communication between dentistry and neurology regarding the overall health optimization of patients with CSVD.

## Author contributions

X.J. Huang and X. Liang contributed equally to the conception and revision of the manuscript. Y.J. Hao and Y.Q. Huang contributed to the experiment design, data acquisition, and manuscript drafting. H.N. Cai and R.N. Chen contributed to data analysis.

## Ethics statement

This study was registered at ClinicalTrials.gov (Identifier: NCT06479915) and approved by the Ethics Committee of Stomatological Hospital Affiliated to Fujian Medical University (No. 2024ECSS027).

## Conflict of interest

None disclosed.

## Appendix Supplementary materials

Image, application 1

Image, image 2 dummy alt text

Image, application 3

## Footnotes

Supplementary material associated with this article can be found in the online version at doi:10.1016/j.identj.2026.109787.
