# Supplementary material for: Association Between cnm-Positive Streptococci and Cerebral Small Vessel Disease: Insights From Oral Health and Microbiome Status
Source: Int Dent J. 2026 Aug 1;76(5):109787. doi: 10.1016/j.identj.2026.109787 (PMC13453468; doi:10.1016/j.identj.2026.109787)
Supplement: Supplementary file 1 [file mmc1.docx]

**Figure S1 Detection of the *cnm* gene in participants' dental plaque samples of via PCR and agarose gel electrophoresis.**

**Table S1 Detailed annotation and cnm gene sequence alignment of discriminating microbial taxa from the phylum to species levels.**
